# Potentially Suitable Habitat for the Pest *Histia rhodope* Based on Its Host Plant *Bischofia polycarpa* and Climatic Factors in China

**DOI:** 10.3390/insects16060627

**Published:** 2025-06-13

**Authors:** Huicong Du, Jingxin Shen, Wenping Luo, Zi Yang, Daizhen Zhang, Xiangbo Kong

**Affiliations:** 1 Jiangsu Provincial Key Laboratory of Coastal Wetland Bioresources and Environmental Protection, School of Wetlands, Yancheng Teachers University, Yancheng 224007, China; duhc@yctu.edu.cn (H.D.); 19983755935@139.com (W.L.); 13385557327@139.com (Z.Y.); 2 Jiangsu Key Laboratory for Bioresources of Saline Soils, Jiangsu Synthetic Innovation Center for Coastal Bio-Agriculture, Yancheng Teachers University, Yancheng 224007, China; 3 School of Hydraulic Engineering, Fujian College of Water Conservancy and Electric Power, Yong’an 366000, China; shenjingxin@fjsdxy.com; 4 Key Laboratory of Forest Protection of the National Forestry and Grassland Administration, Ecology and Nature Conservation Institute, Chinese Academy of Forestry, Beijing 100091, China

**Keywords:** climate change, host plant, insect population, MaxEnt, suitable areas

## Abstract

*Histia rhodope* is a defoliating pest that feeds mainly on the ornamental garden plant *Bischofia polycarpa*. Recently, frequent outbreaks of *H. rhodope* in Southern China have severely affected cityscapes and people’s lives. To provide a predictive early-warning program for the spread risk of *H. rhodope* in China and reduce damage to *B. polycarpa,* we used the MaxEnt model to investigate the potentially suitable spread characteristics of *H. rhodope* and its host *B. polycarpa* under different climate scenarios for the years 2050 and 2070. The results showed that the potentially suitable habitat of *H. rhodope* under the SSP5-8.5 scenario will reach an area of 3174.55 × 10^3^ km^2^ in the 2070s, an increase of 1010 × 10^3^ km^2^ from the current distribution. The potentially suitable habitat of *B. polycarpa* under the SSP5-8.5 scenario will reach 2618.01 × 10^3^ km^2^ in the 2070s (an increase of 464 × 10^3^ km^2^). The potentially suitable habitats of *H. rhodope* and *B. polycarpa* are expected to shift to higher elevations under future climate scenarios. We also identified ten key environmental factors, of which Precipitation of Warmest Quarter (bio18) had the greatest influence on the probability of moth and host plant occurrence. Our results highlight the risk of further expansion of the potentially suitable area for *H. rhodope* and the important role of the host plant in this process, and provide a firm scientific basis for the monitoring and management of *H. rhodope* and *B. polycarpa*.

## Simple Summary

In this study, the MaxEnt model was used to investigate the potentially suitable ranges for the defoliating pest *Histia rhodope* and its main host *Bischofia polycarpa*. The results showed that 8 of the 10 most important climatic factors influencing the distribution of potential habitats are common for both the moth and its host tree. Under different climate scenarios in the 2050s and 2070s, the potentially suitable areas are predicted to increase and spread to high-latitude regions, particularly for *H. rhodope*. These results provide information that can help monitor the potentially suitable areas of *H. rhodope* and improve our understanding of the climate-driven distribution of this species.

## 1. Introduction

Forest pests and climate change are the greatest biotic and abiotic threats to native trees in the 21st century [1,2]. Invasive insects cause more than USD 70 billion in economic losses worldwide each year, and climate change is expected to exacerbate the impact of forest pests and cause significant ecological damage over the next century [3,4]. The Sixth Assessment Report published by the United Nations Intergovernmental Panel on Climate Change (IPCC) in 2023 revealed that with increasing global warming, many species will migrate on a large scale [5,6]. Some pests will also migrate and spread to new habitats [7,8] that were previously too cold for them to survive in, and thus the trend of populations expansion will become more pronounced [9,10]. In addition, the effects of extreme climate conditions on host-plant susceptibility may also play an important role in pest outbreaks, increasing the risk of pest colonization [11,12]. Therefore, global climate change will not only promote the invasion of pests but also change their distribution patterns [13]. Understanding the responses of insects, especially pests, to future climate change will help clarify the factors that influence the geographical distribution of pests [14]. At the same time, it is helpful to formulate strategies for their management based on distribution trends.

**Figure 1:**
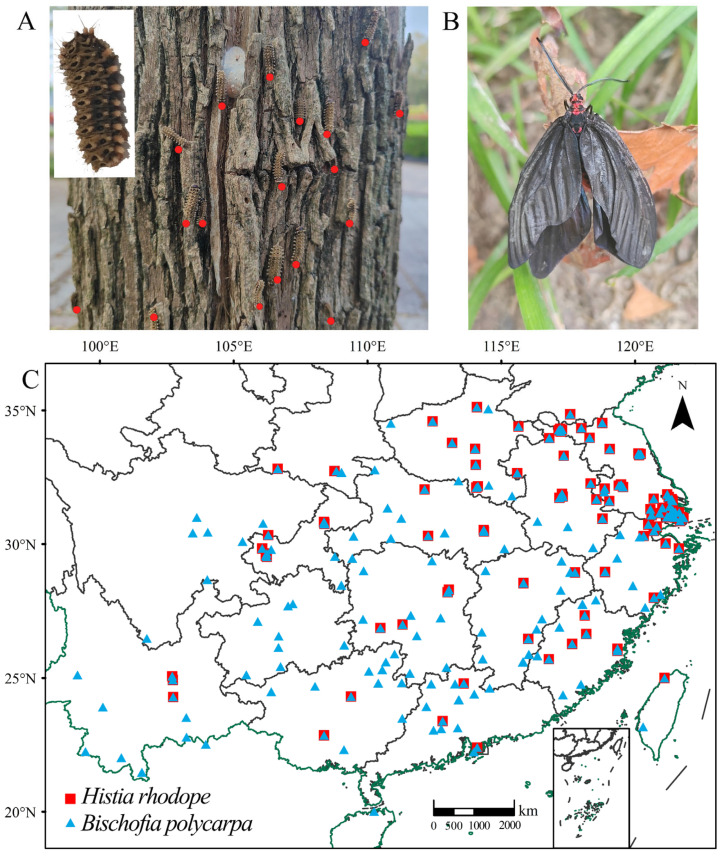
The larvae (**A**) and adults (**B**) of *Histia rhodope* and the geographical distribution of *H. rhodope* and its host plant *Bischofia polycarpa* (**C**). The red dots indicate the positions of the larvae.

The diurnal moth *Histia rhodope* Cramer (Lepidoptera: Zygaenidae) is one of the most destructive and native defoliator pests widely distributed in the central and southern provinces of China [15]. This pest mainly feeds on the street tree *Bischofia polycarpa* (Levl.) and often causes severe damage. The native ornamental plant *B. polycarpa* is the most important tree for landscaping in many cities in China because of the excellent quality of its woods, its beautiful appearance, and its wide distribution [16]. The moth can eat off all the leaves of *B. polycarpa*, leaving only bare branches and leaf veins, severely affecting the photosynthesis of the host plants and thus impairing normal growth and development [16]. Furthermore, the larvae of *H. rhodope* (Figure 1A) fall from these trees by spinning silk, and this habit greatly disturbs the cityscape and human activities in the city [17,18]. Given the severity of the damage caused by *H. rhodope*, it is critical to determine the current and future climatic regions suitable for the survival of this species.

In recent years, there have been many reports about the impact of the moth *H. rhodope* on the urban landscape and residents’ lives in China. According to earlier reports, this pest was mainly distributed in the region south of the Yangtze River [15]. However, in recent years, the pest has also been found in Zaozhuang City (Shandong Province), Luoyang City (Henan Province), and elsewhere [17,19], indicating that the geographical distribution of *H. rhodope* is gradually spreading northward. For example, Yang et al. [20] investigated the influence of the cold resistance of *H. rhodope* on its geographical distribution and found that the moth may be limited by the low temperatures along the 40° latitude. However, under the influence of future climate change, it is not known whether this limit will be exceeded or how the potentially suitable habitat of *H. rhodope* will spread. Predicting suitable areas for species dispersal is an important area of research in ecology, and the dispersal model is an important tool for investigating suitable areas for the geographical distribution of species [21]. The maximum entropy model (MaxEnt) is the most commonly used model for species dispersal. It is characterized by its ease of use, short running time, high accuracy, and stable of prediction results [22,23,24]. Since the MaxEnt model provides good results in terms of assessing potential distributions even with sparse species distribution data [25,26], it has been used to predict suitable areas for various pests [27,28], and the effects of climate change have gradually become an important factor in model prediction. In addition, in studies on the prediction between phytophagous insects and their host plants, the interspecific relationship is used as the boundary condition of the prediction model, which provides greater ecological relevance for the prediction of distributions [29]. Therefore, the distribution of suitable areas for the *H. rhodope* moth and its main host plant *B. polycarpa* was investigated and analyzed in this study.

In summary, the MaxEnt model was used to predict the potential spread of the pest and its major host plant in China. The objectives of this study were as follows: (1) to analyze the current potential distribution of *H. rhodope* and *B. polycarpa* in China; (2) to investigate the main environmental factors affecting the distribution of *H. rhodope* and *B. polycarpa*; and (3) to predict the distribution of potential habitats of *H. rhodope* and its host plant *B. polycarpa* in the 2050s and 2070s under different climate scenarios.

## 2. Materials and Methods

### 2.1. Collection and Processing of Distribution Data

The potential geographical distributions of *H. rhodope* and *B. polycarpa* in China were estimated using data from the following sources: (1) the literature and news reports, and (2) field surveys and collections. We conducted a field survey of the areas where the adults and larvae of *H. rhodope* (Figure 1A) are distributed and identified 121 distribution sites (Supplementary Table S1). We also obtained 372 distribution locations of the host plants *B. polycarpa* (Supplementary Table S2), mainly from two sources, the GBIF database (Global Biodiversity Information Facility: https://www.gbif.org/, accessed on 20 March 2025) and field surveys. The research data are stored at the School of Wetlands, Yancheng Teachers University. In order to ensure the simulation was accurate and reduce errors or deviations caused by spatial autocorrelation, the buffer analysis in ArcGIS v10.4.1 was used to screen the distribution data [30], and the buffer radius was set to 5 km. Finally, the data used in further analysis consisted of 102 distribution points for *H. rhodope* and 287 distribution points for *B. polycarpa* (Figure 1C).

Based on the current geographical distribution data of *H. rhodope* and *B. polycarpa*, we predicted their potentially suitable distributions in the present, 2050s, and 2070s. The environmental data required for model prediction consisted of 19 environmental factors (Supplementary Table S3) obtained from the WorldClim database (www.worldclim.org/, accessed on 23 March 2025), with a spatial resolution of 2.5 arc minutes. The addition of socio-economic factors as part of the Sixth International Coupled Model Intercomparison Program (CMIP6) made the simulation results more scientific and accurate [31,32]. Therefore, in this study, the climate factors suitable for China in the CMIP were selected for the future environmental variable data, including three typical common socio-economic pathways: SSP1-2.6 (Sustainable Development Pathway), SSP2-4.5 (General Development pathway), and SSP5-8.5 (Rapid Development pathway). To improve the model’s accuracy and avoid its transition adjustment, 19 environmental factors were extracted to the geographical distribution points of *H. rhodope* and *B. polycarpa* using ArcGIS. The data were exported for Pearson’s correlation analysis using SPSS 25 software [33]. There are problems of correlation among the 19 environmental factors [34]: this redundant information may affect the accuracy and significance of the prediction results. Therefore, in this study, a correlation analysis of the environmental factors based on the geographical distribution sites of *H. rhodope* and *B. polycarpa* was conducted, and redundant information was eliminated. If the absolute value of the correlation coefficient was >0.8 and a correlation was significant (*p* < 0.01), the factors with a higher contribution rate were retained, and the verified environmental factors were used for further analysis [35,36] (Supplementary Tables S4 and S5).

### 2.2. Optimization and Construction of the MaxEnt Model

MaxEnt v10.4.1 software was used to predict the current geographical distribution of *H. rhodope* and *B. polycarpa,* as well as their future geographical distribution under different SSP scenarios in the 2050s and 2070s. The distribution data of *H. rhodope* and *B. polycarpa* were imported into MaxEnt software, together with the data regarding the main environmental variables [37]. For the training data, 75% of the distribution points were randomly selected. For the test data, 25% of the distribution points were selected. Validation and prediction models were created for the training data and the test data, respectively. The jackknife test was employed to determine the weight of each variable, and a response curve of the selected environmental variables was created. The output format was “Logistic”, with the repetition of the training run set to “10”, the maximum number of iterations set to “5000”, and the repetition mode set to “Subsample” [36]. The area under the receiver operating characteristic curve (area under the curve, AUC) was used to evaluate the simulation results for the model. According to a series of different dichotomies, a curve was drawn, with the false-positive rate (the ratio that does not exist but is predicted) and the true-positive rate (the ratio that actually exists and is predicted) serving as horizontal and vertical coordinates, respectively [38].

### 2.3. Analysis of the Potential Distribution Under Current and Predicted Climate Change

The output of the MaxEnt model was the probability of *H. rhodope* and *B. polycarpa* occurring in China, and the data were in ASCII format. First, the data were converted into raster format using a format conversion tool (ArcGIS ArcToolbox), and the results were displayed in ArcGIS [30]. Then, the distribution probabilities for the moth and its host plant in China were determined using the “Extraction and Analysis” function. The natural discontinuity points classification method was used in ArcGIS to reclassify the predicted distribution results, and the different degrees of potentially suitable areas were represented using different colors. The Kappa (k) value of the classification criteria (Table 1) was developed by considering the results of similar insects [39] and plants [40] and our analysis of the current distribution. ArcGIS was used to calculate the ratio of the number of grids in each suitable habitat to the total number of grids, and the distribution area for the moth and its host was calculated based on their grid proportions for the land area of China [33].

### 2.4. Analysis of Centroid Migration Under Predicted Climate Change

To investigate the direction of changes in the potential distribution areas of the moth and its host plant under the current and future different SSP scenarios, we summarized the distribution as a single point, namely, the centroid of the potentially suitable area [41,42]. The prediction results were converted into binary files with a threshold of MTSPS (maximum training sensitivity plus specificity) using the SDM (species distribution model) toolbox in ArcGIS. The “Distribution Changes Between Binary SDMs” and “Centroid Changes (Lines)” SDM Toolboxes were used to determine the range expansion, range contraction, and centroid changes for *H. rhodope* and *B. polycarpa* under different future SSP scenarios [43].

## 3. Results

### 3.1. Screening and Importance of Key Environmental Factors

The number of key environmental factors affecting the potentially suitable distribution of *H. rhodope* and *B. polycarpa* is 10, of which 9 factors are shared by both species; these include the Annual Mean Temperature (bio1), Mean Diurnal Range (bio2), Temperature Seasonality (bio4), Mean Temperature of Wettest Quarter (bio8), Mean Temperature of Warmest Quarter (bio10), Precipitation of Wettest Month (bio13), Precipitation of Driest Month (bio14), Precipitation Seasonality (bio15), Precipitation of Warmest Quarter (bio18). Isothermality (bio3) and Min Temperature of Coldest Month (bio6), which are only available for *H. rhodope* and *B. polycarpa*, respectively. The cumulative contributions of the top ten environmental factors to the MaxEnt model for *H. rhodope* and *B. polycarpa* were 95.4% and 95.9%, respectively (Table 2). The contributions of bio4 and bio18 were the highest for both the moth and the host plant, with overall contributions of 72.1% and 77.2%, respectively.

**Figure 2:**
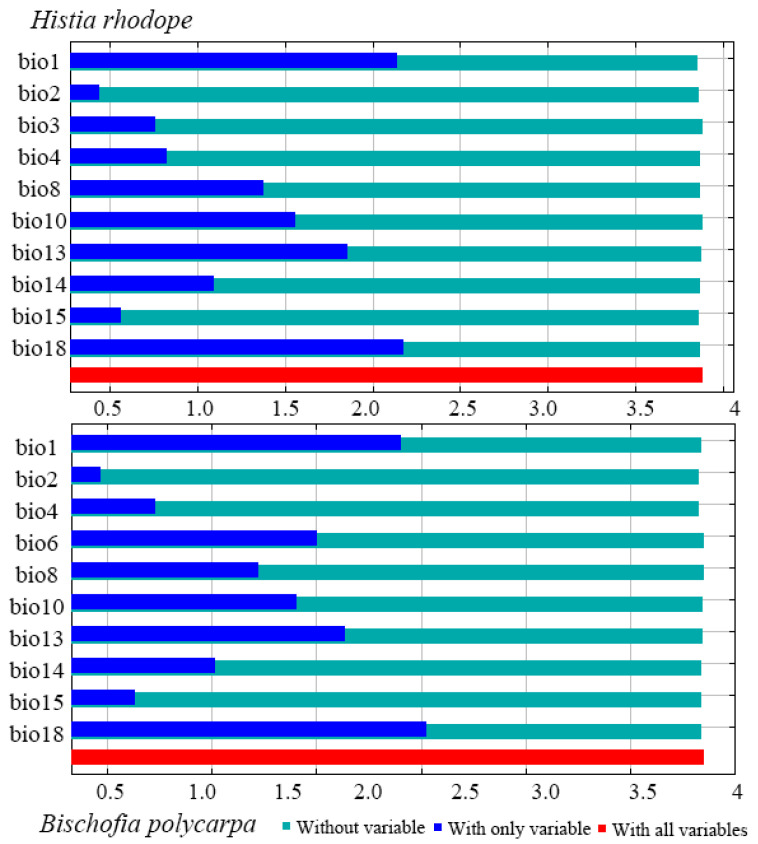
Regularized training gain of environmental factors for predicting the potential geographical distributions of *Histia rhodope* and *Bischofia polycarpa* by Jackknife.

The jackknife method was used to analyze the ten most environmental factors. The results show that when only one variable was used in the analysis of the regularized training gain (Figure 2), test gain and AUC (Supplementary Figure S1), Precipitation of Warmest Quarter (bio18) and Annual Mean Temperature (bio1) are the two variables with the highest gain values for the predicted distribution of suitable areas for *H. rhodope* and *B. polycarpa*, while the values for the Mean Diurnal Range (bio2) and Precipitation Seasonality (bio15) are the lowest. In three different gain analyses, there was some similarity in the differences in gain scores for the same environmental factors adopted for analyzing *H. rhodope* and *B. polycarpa*.

### 3.2. Prediction of the Distribution of Suitable Habitats for H. rhodope and B. polycarpa Under Current Conditions

**Figure 3:**
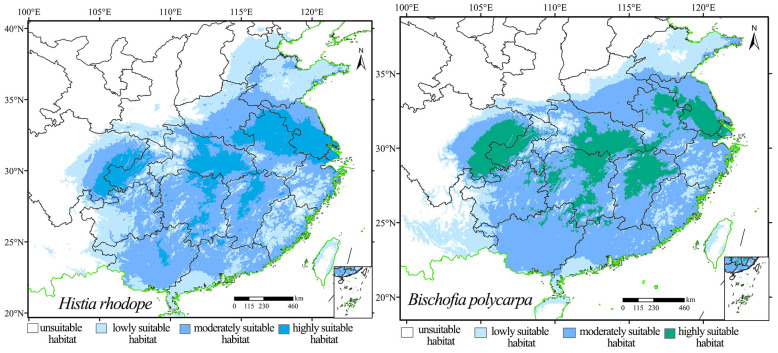
Potential distribution of *Histia rhodope* (**left**) and *Bischofia polycarpa* (**right**) in different areas of China under current climatic conditions. The maps in the bottom-right corner show the South China Sea and Taiwan, as indicated below.

**Figure 4:**
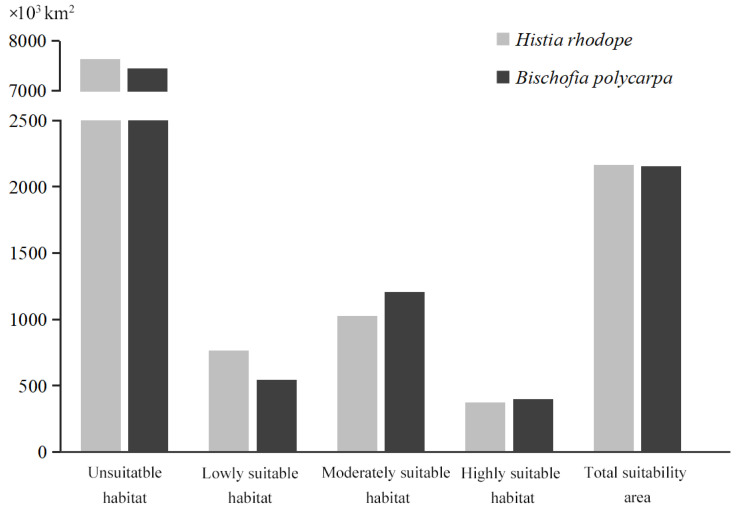
Bar chart for the current suitable habitat area predicted for *Histia rhodope* and *Bischofia polycarpa* based on the MaxEnt model.

The AUC values for the model predicting the current population distribution of *H. rhodope* and *B. polycarpa* were 0.972 and 0.953 (i.e., >0.9), respectively (Supplementary Figure S2). These results indicate that the MaxEnt models constructed in this study provide reliable results for predicting the suitable distribution areas of *H. rhodope* and *B. polycarpa*. Maps of the current distribution of suitable habitats for *H. rhodope* and *B. polycarpa* were generated. The results showed that *H. rhodope* and *B. polycarpa* are mainly distributed in the southeastern Provinces of China (Figure 3). There are almost no suitable habitats for *H. rhodope* in Yunnan Province, while there are areas of low suitability for *B. polycarpa*; the opposite picture is in northern China, where there are no suitable habitats for *B. polycarpa* in Hebei Province, while for *H. rhodope* there are the areas of low suitability. The predicted total distribution area of *H. rhodope* was 2164.31 × 10^3^ km^2^. The area of highly, moderately, and poorly suitable habitats was 373.54 × 10^4^, 1027.17 × 10^3^, and 763.60 × 10^3^ km^2^, respectively (Figure 4). The total suitable area for *B. polycarpa* was 2154.16 × 10^3^ km^2^, with the areas of highly, moderately, and poorly suitable habitats reaching 400.13 × 10^3^, 1207.89 × 10^3^, and 546.13 × 10^3^ km^2^, respectively. With the exception of the less suitable habitat, the areas of highly suitable habitat and moderately suitable habitat for *B. polycarpa* were slightly larger than those for *H. rhodope*. The total suitable areas for *H. rhodope* and *B. polycarpa* were similar.

### 3.3. Prediction of the Distribution of the Suitable Habitats for H. rhodope and B. polycarpa in the Future

**Figure 5:**
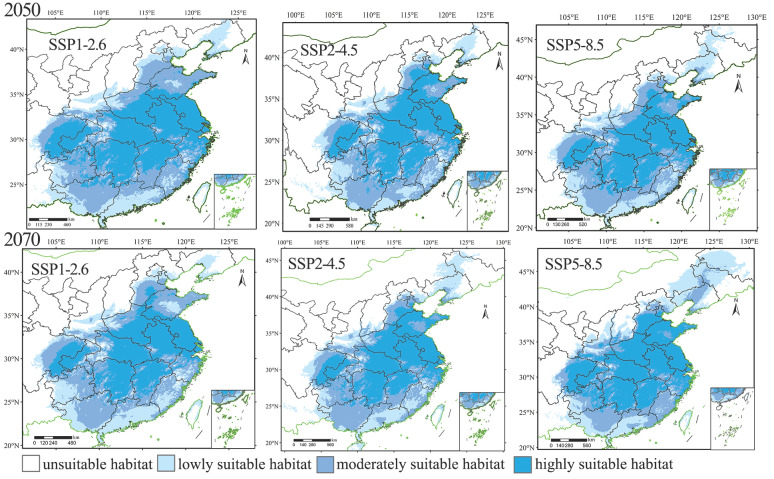
Potential distribution of *Histia rhodope* under different climate scenarios in the 2050s and 2070s.

**Figure 6:**
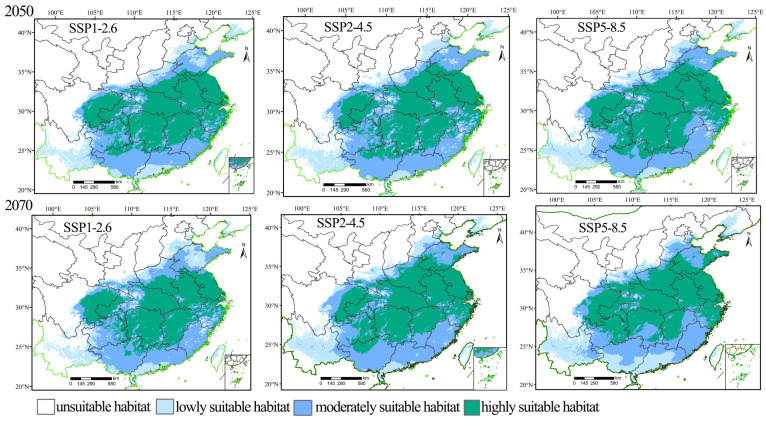
Potential distribution of *Bischofia polycarpa* under different climate scenarios in the 2050s and 2070s.

Under the three climate scenarios (SSP1-2.6, SSP2-4.5, and SSP5-8.5) in the 2050s and 2070s, the potentially suitable habitat area for *H. rhodope* and its host *B. polycarpa* was mainly in Southeastern China (Figure 5 and Figure 6, respectively), with the most notable changes in Liaodong Peninsula, Hebei Province, and Yunnan Province. Under scenario SSP1-2.6, a less suitable habitat for *H. rhodope* began to appear in the Liaodong Peninsula in the 2050s and 2070s. Under scenarios SSP2-4.5 and SSP5-8.5, a moderately suitable habitat gradually appears in the Liaodong Peninsula. Under the three climate scenarios in the 2050s and 2070s, the moderately and highly suitable habitat in the Hebei Provine is much larger than that of current. Under different climate scenarios in the 2050s and 2070s, the less suitable habitat area for *B. polycarpa* will gradually expand in the Liaodong Peninsula and Hebei Province. In addition, the moderately suitable habitats of *B. polycarpa* will also gradually expand in Yunnan Province. The changes in the distribution of each potential suitable habitat for *H. rhodope* are generally larger than those of the potential suitable habitat for *B. polycarpa*.

The area of highly suitable habitats for *H. rhodope* and *B. polycarpa* will increase significantly compared to the current range under the different SSP scenarios (Supplementary Table S6), and the areas of the least and moderately suitable habitats for *H. rhodope* and *B. polycarpa* will decrease. The highest increase for *H. rhodope* and *B. polycarpa* in highly suitable habitats is expected under the SSP5-8.5 scenario in the 2070s, with increases of 970 × 10^3^ km^2^ and 822 × 10^3^ km^2^, respectively. The total suitable area of the two species will also increase in the future under various scenarios. Under the scenario SSP5-8.5 in 2070, the area of the potential distribution of *H. rhodope* will increase the most, rising by 1010 × 10^3^ km^2^. Under the scenario SSP5-4.5 in 2070, the area of potential distribution of *B. polycarpa* will increase the most, by 621 × 10^3^ km^2^.

### 3.4. Migration of the Centroids of Suitable Habitats

**Figure 7:**
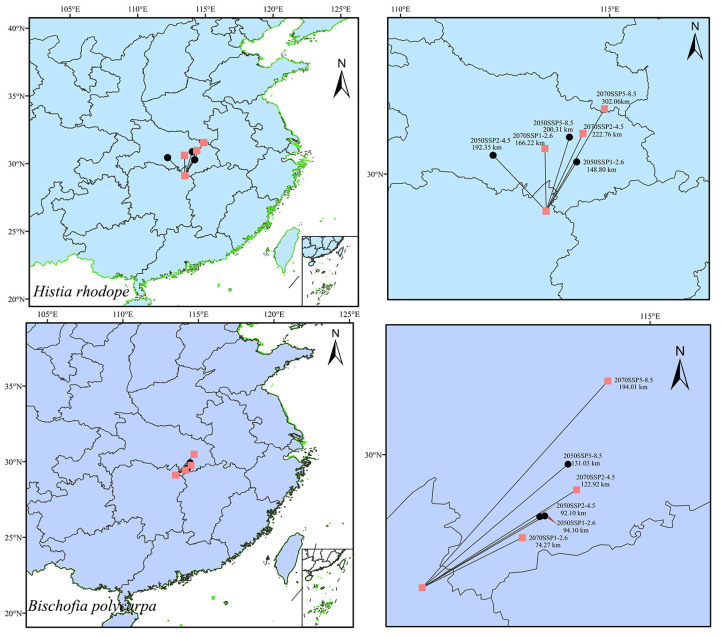
Centroid migration for *Histia rhodope* and *Bischofia polycarpa* in the 2050s and 2070s under three climate scenarios.

The predictions of the centroid migration of *H. rhodope* and *B. polycarpa* under the three climate scenarios (SSP1-2.6, SSP2-4.5, and SSP5-8.5) in the 2050s and 2070s are shown in Figure 7. The direction and distance of the migration of the centroids for *H. rhodope* and *B. polycarpa* are not exactly the same under the different scenarios in the 2050s and 2070s, but the directions of centroids are both towards high-latitude regions. All the centroids for *B. polycarpa* move northeastward, and the centroids for *H. rhodope* shift radially northward. Under different scenarios in the 2050s and 2070s, the centroid transfer distances for *H. rhodope* are 148.80 km (2050SSP1-2.6), 192.35 km (2050SSP2-4.5), 200.31 km (2050SSP5-8.5), 166.22 km (2070SSP1-2.6), 222.76 km (2070SSP2-4.5), and 302.06 km (2070SSP5-8.5); the centroid transfer distances for *B. polycarpa* are 94.10 km (2050SSP1-2.6), 92.10 km (2050SSP2-4.5), 131.03 km (2050SSP5-8.5), 74.27 km (2070SSP1-2.6), 122.92 km (2070SSP2-4.5), and 194.01 km (2070SSP5-8.5). The centroids transfer distances of *H. rhodope* are generally larger than that of *B. polycarpa.*

## 4. Discussion

The MaxEnt model used nineteen environmental factors to predict the geographical distribution of *H. rhodope* and *B. polycarpa*. After the correlated environmental factors were eliminated, both *H. rhodope* and *B. polycarpa* were assigned ten core environmental factors. The core environmental factors and their contribution to the distribution of *H. rhodope* and its main host plant *B. polycarpa* showed a high degree of similarity, which was related to the feeding habits of *H. rhodope*. The similarity of the current potentially suitable habitat distribution for *H. rhodope* and that of its host also confirmed this conclusion. Among all the environmental factors analyzed, Precipitation of Warmest Quarter (bio18) accounted for the highest distribution of potentially suitable habitats for the moth and its host, indicating that this variable has more unique information and is more important with respect to species distribution [44,45]. In addition, low winter temperatures are also a very important environmental factor that significantly influences the extent of insects’ geographic range [46]. For an insect to be able to colonize a geographical area, a sufficient number of individuals that can survive the winter are needed [47], and insects’ ability to withstand low temperatures allows them to survive the winter and reproduce rapidly once the cold season is over [20]. In most of its distribution areas in China, there are four generations of *H. rhodope* per year, and the old larvae form cocoons in bark cracks, leaves on the ground, or wall corners to overwinter [17]. Yang et al. [17] investigated the overwintering overcooling point and found that the cold tolerance of *H. rhodope* is subject to seasonal variation and that the metabolic changes in larvae are related to the changes in temperature and humidity in winter. Temperature Seasonality (bio4), Mean Temperature of Wettest Quarter (bio8), Mean Temperature of Warmest Quarter (bio10), Precipitation Seasonality (bio15), and Precipitation of Warmest Quarter (bio18), which we investigated, are all seasonal environmental factors.

In this study, the potentially suitable areas for *H. rhodope* and its main host *B. polycarpa* were predicted under current and future climate scenarios. The results showed that the suitable habitat area for this moth is large, with the current suitable habitat area being 2164.31 × 10^3^ km^2^, and the suitable habitat area for its host *B. polycarpa* being 2154.16 × 10^3^ km^2^. The distribution area of the host plant essentially covered the area of the moth. By estimating its overwintering potential, Yang et al. [20] concluded that the current distribution of the suitable area for *H. rhodope* is limited by low temperatures along the 40° north latitude and that it is possible to extend its range further into cooler areas, findings consistent with our current prediction of the potential suitable distribution. In the future, under different SSP scenarios in the 2050s and 2070s, the area of suitable habitat for *H. rhodope* will increase greatly compared to the distribution today. Under the scenario SSP5-8.5 in the 2070s, the total suitable area of the moth is projected to reach 3174.55 × 10^3^ km^2^ (an increase of 1010 × 10^3^ km^2^, with a growth rate of 46.67%). The centroid will shift to higher latitudes, and the suitable habitat is predicted to emerge in the Liaodong Peninsula in China, indicating that the potentially suitable habitat for *H. rhodope* will exceed the northern limit of 40° north latitude for overwintering under the climate-warming conditions mentioned by Yang et al. [20]. Under different future climate scenarios, a potentially suitable habitat area for *B. polycarpa* is also projected to gradually appear in the Liaodong Peninsula, and the centroid will also shift to the high-latitude area, showing a certain degree of synchronization with *H. rhodope*. This is even if the direction of change in distribution is similar, and the potential suitable area in the Liaodong Peninsula is much larger for *H. rhodope* than that of its host plant. However, the moth will not be able to establish itself in new suitable areas if host trees are not available. This suggests that it is not wise to plant *B. polycarpa* in this or similar regions because (1) the conditions are less favorable for growth and (2) there is a high risk of infestation by the moth. There are provinces where the trend is opposite, with the area potentially suitable for the host plant being larger than that for the moth. This suggests that planting the host plant has a lower risk. In addition, although *H. rhodope* feeds mainly on *B. polycarpa*, it can also feed on *Bischofia javanica* Blume during large outbreaks or food shortages. Apart from the sufficient food plant, *H. rhodope* can also produce and store cyanide in its body to ward off any predators. Therefore, the population growth of Zygaenidae species can easily become uncontrolled in the wild. Similar cases have been reported regarding *Achelura yunnanensis* by Horie & Xue on cherry trees in Central Yunnan Province [48].

Similar studies involving the use of MaxEnt have shown that many insects, such as *Helicoverpa zea* Boddie [49] and *Anoplophora glabripennis* Motschulsky [50], will spread to higher latitudes under future global climate change scenarios. Because of global warming, the environmental temperature is gradually approaching the optimal level for the development of many pests. The severity of most pest damage is expected to increase at mid and low latitudes, reducing the thermal constraints on population dynamics [51]. *Histia rhodope* is no exception. Since last year, there have been many reports from the cities of Wuhan and Xiangyang in Hubei Province that *H. rhodope* has caused problems for pedestrians; this has gradually attracted a great amount of attention from workers in the landscaping and greening industry. Therefore, to prevent further outbreaks, it is necessary to closely monitor the spread of the suitable areas for *H. rhodope* and its hosts in real time.

However, this study has some limitations that weaken the predictions made by the model. The MaxEnt model describes the theoretical niche of a species, not the actual niche, and its prediction accuracy depends on the quantity and quality of available data and the suitability of the environmental factors used [52,53,54]. The conditions of Yunnan Province should not be favorable for *H. rhodope*, while are of low suitability for the host plant, but Figure 1 shows that both the host plant and the moth are present there. The moth can be present, but due to unfavorable conditions, the population might not develop as fast as in other regions. Certain limitations can be avoided by combining the real-time updating of distribution data with predictions made by other models. In addition, *H. rhodope* is highly resistant to cold [20] and usually hibernates under leaf litter on the surface or in the crevices of buildings where the temperature is higher than the air temperature. Therefore, its survival rate at high latitudes should be greater than the theoretical estimates, and the distribution of suitable areas based on modeling should also be greater. In addition, the distribution of *H. rhodope* is influenced by its ability to fly, human factors, geographical barriers, and species interaction [55,56,57]. How these factors are incorporated into model predictions remains to be investigated.

To prevent further outbreaks of *H. rhodope*, management strategies for response should be formulated in addition to predicting suitable habitats. For example, plant quarantines should be strengthened. Given the further spread of *H. rhodope* and *B. polycarpa* in the Liaodong Peninsula, Hebei Province and Yunnan Province noted in this study, we suggest paying attention to plant quarantine regulations when introducing *B. polycarpa* as a roadside tree, especially since *B. polycarpa* originates from areas heavily infested by *H. rhodope*. If a moth infestation occurs on a large scale, highly effective and low-pollutant chemical control agents can be used [58]. When *B. polycarpa* is planted as a street tree in urban areas, it can be mixed and planted in strips or blocks with other insect-resistant tree species such as *Robinia pseudoacacia*. In the fall and winter, the tree trunks should be treated with bleaching agents to increase their insect resistance [59], and the dead branches and fallen leaves should be removed in time to prevent the formation of cocoons and overwintering inside [60]. In addition, research on the sex pheromones of *H. rhodope* is still nascent, and the development of these bioactive and environmentally friendly biological agents must also be urgently addressed.

## 5. Conclusions

We used the MaxEnt model to analyze the potential suitable habitat for *H. rhodope* and its host plant *B. polycarpa* under current and future different climate scenarios. The results showed that the model was a good fit, and ten key environmental factors were selected, of which Precipitation of Warmest Quarter (bio18) had the greatest impact on the distribution of the moth and its host. Under the current climate conditions, the moth and its host plant are widely distributed in different Provinces in China. Under different SSP scenarios in the 2050s and 2070s, the potential suitable habitats of the two species are expected to expand significantly to high latitudes. The centroids of the suitable habitats for *H. rhodope* and *B. polycarpa* will also migrate northward to varying degrees. In this study, only climate and the host plant were considered for modeling. In the future, more variables and models should be integrated to improve the accuracy of prediction. Ultimately, these results will allow a more accurate assessment of the risk of spread and outbreaks of this pest, improving our capacity for early prevention and response.

## Figures and Tables

**Table 1:** Classification criteria for the suitable habitat area for *Histia rhodope* and *Bischofia polycarpa*.

Species	Unsuitable Habitat	Lowly Suitable Habitat	Moderately Suitable Habitat	Highly Suitable Habitat
*Histia rhodope*	k < 0.08	0.08 ≤ k < 0.27	0.27 ≤ k < 0.55	k > 0.55
*Bischofia polycarpa*	k < 0.20	0.20 ≤ k < 0.40	0.40 ≤ k < 0.60	k > 0.60

**Table 2:** Percentage contributions of the ten most important environmental factors to the MaxEnt model for *Histia rhodope* and *Bischofia polycarpa*.

Environmental Factors and Description	Percent Contribution	
	(*H. rhodope*)/%	(*B. polycarpa*)/%
Bio18: Precipitation of Warmest Quarter	52.1	58.2
Bio4: Temperature Seasonality	20	19
Bio15: Precipitation Seasonality	4.8	6.6
Bio14: Precipitation of Driest Month	4.5	3.9
Bio13: Precipitation of Wettest Month	3.9	3.7
Bio10: Mean Temperature of Warmest Quarter	2.2	1.4
Bio8: Mean Temperature of Wettest Quarter	5.7	0.5
Bio1: Annual Mean Temperature	0.6	0.4
Bio2: Mean Diurnal Range	0.8	0.1
Bio6: Min Temperature of Coldest Month.	-	2
Bio3: Isothermality (Bio2/Bio7) (×100).	0.8	-
Total percent contribution	95.4	95.9

## Data Availability

The raw data supporting the conclusions of this article will be made available by the authors upon request.

## Supplementary Materials

The following supplementary information can be downloaded at: https://www.mdpi.com/article/10.3390/insects16060627/s1. Figure S1: Jackknife test of the AUC and test gain values for key bioclimatic factors; Figure S2: AUC values of the MaxEnt model applicability test for *Histia rhodope* (left) and *Bischofia polycarpa* (right); Table S1: Original distribution data of *Histia rhodope*; Table S2: Original distribution data of *Bischofia polycarpa*; Table S3: Environmental variables used to simulate suitable areas for *Histia rhodope* and *Bischofia polycarpa* and their contribution rates; Table S4: Pearson correlation matrix of environmental factors based on the geographic distribution of *Histia rhodope*; Table S5: Pearson correlation matrix of environmental factors based on the geographic distribution of *Bischofia polycarpa*; Table S6. Predicted area of suitable habitats for *Histia rhodope* and *Bischofia polycarpa* under different climate scenarios.
