# Supplementary material for: Potentially Suitable Habitat for the Pest Histia rhodope Based on Its Host Plant Bischofia polycarpa and Climatic Factors in China
Source: Insects. 2025 Jun 13;16(6):627. doi: 10.3390/insects16060627 (PMC12194395; doi:10.3390/insects16060627)
Supplement: Supplementary file 1 [file insects-16-00627-s001.zip › Figures S1-S2.pdf]

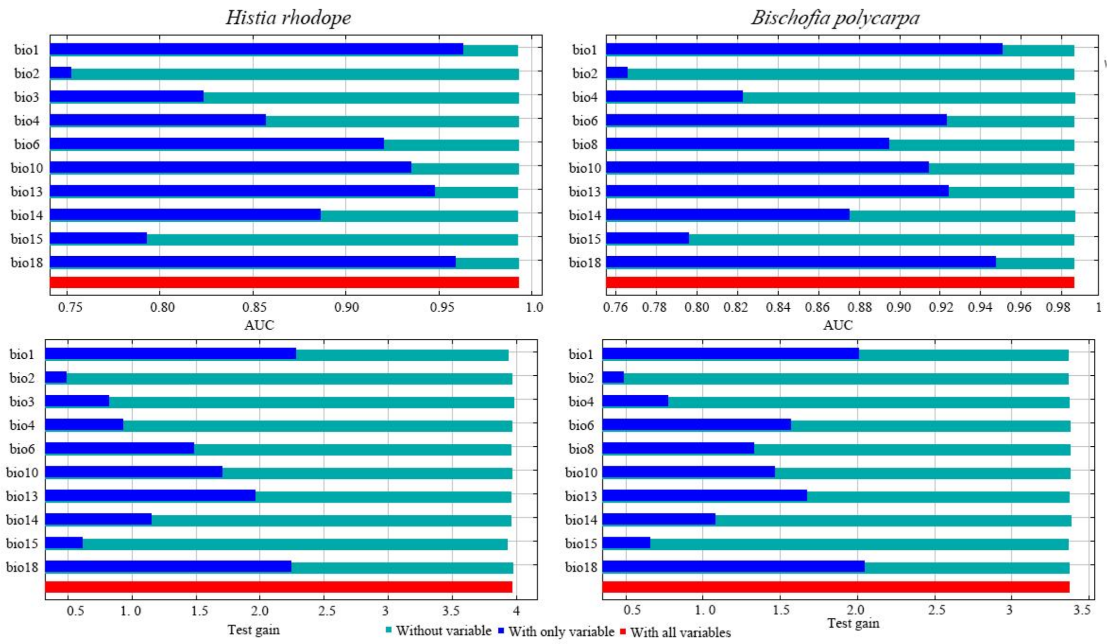


**Figure S1:** Jackknife test of the AUC and test gain values for core bioclimatic factors.

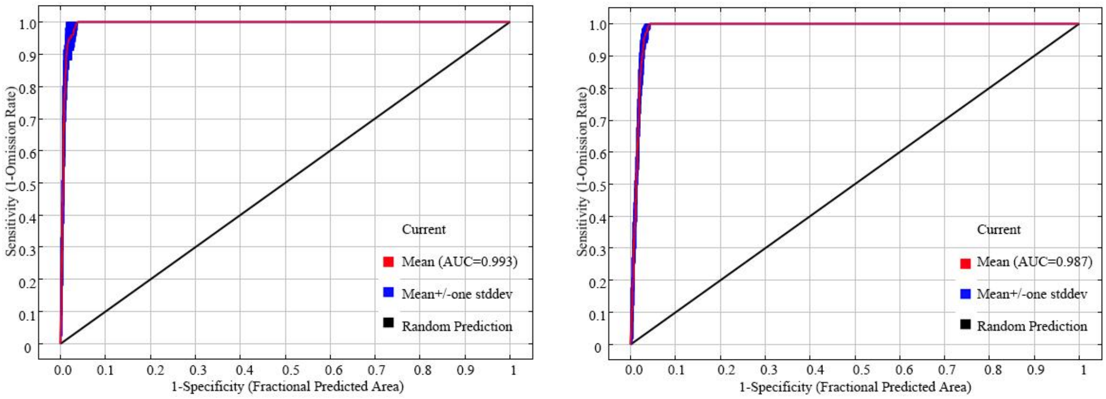


**Figure S2:** AUC values of the MaxEnt models applicability test for *Histia rhodope* (left) and *Bischofia polycarpa* (right).
