# Supplementary material for: Potentially Suitable Habitat for the Pest Histia rhodope Based on Its Host Plant Bischofia polycarpa and Climatic Factors in China
Source: Insects. 2025 Jun 13;16(6):627. doi: 10.3390/insects16060627 (PMC12194395; doi:10.3390/insects16060627)
Supplement: Supplementary file 1 [file insects-16-00627-s001.zip › Tables S3-S6.pdf]

Table S3: Environmental variables used to simulate suitable area for *Histia rhodope* and *Bischofia polycarpa* and their contribution rates.

Environmental factors and description	Percent contribution (*H. rhodope*) / %	Percent contribution (*B. polycarpa*) / %
Bio1：Annual Mean Temperature.	0.6	0.4
Bio2：Mean Diurnal Range.	0.8	0.1
Bio3：Isothermality (Bio2 /Bio7) (*100).	0.8	0.2
Bio4：Temperature Seasonality (standard deviation *100).	20	19
Bio5：Max Temperature of Warmest Month.	0.2	0.4
Bio6：Min Temperature of Coldest Month.	1.9	2
Bio7：Temperature Annual Range (Bio5-Bio6).	0.4	0.2
Bio8：Mean Temperature of Wettest Quarter.	5.7	0.5
Bio9：Mean Temperature of Driest Quarter.	0.4	1
Bio10：Mean Temperature of Warmest Quarter.	2.2	1.4
Bio11：Mean Temperature of Coldest Quarter.	1.5	0.7
Bio12：Annual Precipitation.	0.1	0.1
Bio13：Precipitation of Wettest Month.	3.9	3.7
Bio14：Precipitation of Driest Month.	4.5	3.9
Bio15：Precipitation Seasonality (Coefficient of Variation).	4.8	6.6
Bio16：Precipitation of Wettest Quarter.	0.1	0
Bio17：Precipitation of Driest Quarter.	0	0
Bio18：Precipitation of Warmest Quarter.	52.1	58.2
Bio19：Precipitation of Coldest Quarter.	0.1	0

Table S4: Pearson correlation matrix of environmental factors based on the geographic distribution of *Histia rhodope*

	bio01	bio02	bio03	bio04	bio05	bio06	bio07	bio08	bio09	bio10	bio11	bio12	bio13	bio14	bio15	bio16	bio17	bio18
bio02	-0.422**																	
bio03	0.094	0.616**																
bio04	-0.630**	0.267**	-0.577**															
bio05	0.534**	-0.168	-0.466**	0.273**														
bio06	0.919**	-0.595**	0.123	-0.822**	0.249*													
bio07	-0.859**	0.514**	-0.352**	0.957**	0.242*	-0.880**												
bio08	-0.086	-0.07	-0.432**	0.490**	0.305**	-0.239*	0.389**											
bio09	0.804**	-0.535**	0.091	-0.700**	0.201*	0.853**	-0.756**	-0.244*										
bio10	0.565**	-0.281**	-0.536**	0.281**	0.959**	0.278**	0.193	0.403**	0.255**									
bio11	0.924**	-0.426**	0.311**	-0.876**	0.196*	0.977**	-0.883**	-0.297**	0.849**	0.214*								
bio12	0.819**	-0.449**	0.054	-0.603**	0.367**	0.815**	-0.637**	-0.397**	0.730**	0.378**	0.806**							
bio13	0.625**	0.004	0.293**	-0.431**	0.307**	0.526**	-0.376**	-0.335**	0.432**	0.290**	0.579**	0.756**						
bio14	0.513**	-0.567**	-0.282**	-0.240*	0.311**	0.519**	-0.367**	-0.227*	0.635**	0.392**	0.463**	0.668**	0.258**					
bio15	-0.364**	0.666**	0.431**	0.158	-0.260**	-0.432**	0.305**	0.093	-0.476**	-0.320**	-0.340**	-0.511**	0.103	-0.774**				
bio16	0.722**	-0.167	0.276**	-0.584**	0.269**	0.680**	-0.549**	-0.393**	0.580**	0.254*	0.718**	0.876**	0.936**	0.369**	-0.074			
bio17	0.512**	-0.506**	-0.261**	-0.211*	0.352**	0.491**	-0.319**	-0.306**	0.585**	0.418**	0.444**	0.723**	0.322**	0.968**	-0.778**	0.437**		
bio18	0.600**	-0.194	0.282**	-0.565**	0.071	0.602**	-0.569**	-0.061	0.456**	0.118	0.629**	0.680**	0.773**	0.12	0.064	0.825**	0.147	
bio19	0.594**	-0.466**	-0.177	-0.291**	0.377**	0.559**	-0.375**	-0.333**	0.678**	0.432**	0.531**	0.772**	0.425**	0.957**	-0.712**	0.522**	0.972**	0.202*

Note: ** indicates a significant correlation at the 0.01 level, and * indicates a significant correlation at the 0.05 level.

Table S5: Pearson correlation matrix of environmental factors based on the geographic distribution of *Bischofia polycarpa*

	bio01	bio02	bio03	bio04	bio05	bio06	bio07	bio08	bio09	bio10	bio11	bio12	bio13	bio14	bio15	bio16	bio17	bio18
bio02	-0.046																	
bio03	0.387**	0.678**																
bio04	-0.614**	-0.141*	-0.806**															
bio05	0.543**	-0.094	-0.326**	0.287**														
bio06	0.921**	-0.209**	0.438**	-0.803**	0.253**													
bio07	-0.603**	0.153**	-0.612**	0.949**	0.307**	-0.843**												
bio08	0.125*	-0.132*	-0.185**	0.221**	0.273**	0.003	0.150*											
bio09	0.892**	-0.047	0.478**	-0.725**	0.305**	0.896**	-0.712**	-0.033										
bio10	0.578**	-0.236**	-0.384**	0.287**	0.960**	0.298**	0.241**	0.381**	0.333**									
bio11	0.927**	0.018	0.616**	-0.864**	0.212**	0.971**	-0.838**	-0.028	0.916**	0.235**								
bio12	0.714**	-0.173**	0.172**	-0.459**	0.398**	0.702**	-0.469**	-0.336**	0.690**	0.394**	0.675**							
bio13	0.659**	0.064	0.345**	-0.485**	0.292**	0.620**	-0.448**	-0.235**	0.615**	0.285**	0.642**	0.857**						
bio14	0.311**	-0.357**	-0.347**	0.134*	0.494**	0.220**	0.058	-0.299**	0.347**	0.517**	0.150*	0.630**	0.333**					
bio15	-0.026	0.453**	0.500**	-0.299**	-0.349**	0.006	-0.200**	0.166**	-0.045	-0.363**	0.102	-0.291**	0.175**	-0.730**				
bio16	0.718**	0.022	0.383**	-0.577**	0.276**	0.709**	-0.543**	-0.277**	0.694**	0.265**	0.725**	0.906**	0.969**	0.365**	0.106			
bio17	0.297**	-0.287**	-0.305**	0.135*	0.492**	0.195**	0.082	-0.363**	0.315**	0.499**	0.138*	0.657**	0.364**	0.975**	-0.733**	0.394**		
bio18	0.577**	-0.085	0.429**	-0.654**	-0.051	0.671**	-0.688**	0.026	0.562**	0.017	0.669**	0.650**	0.793**	-0.024	0.335**	0.804**	-0.013	
bio19	0.412**	-0.216**	-0.213**	0.041	0.543**	0.292**	0.015	-0.364**	0.437**	0.539**	0.253**	0.731**	0.476**	0.957**	-0.641**	0.507**	0.962**	0.062

Note: ** indicates a significant correlation at the 0.01 level, and * indicates a significant correlation at the 0.05 level.

Table S6. Predicted area of suitable habitats for *Histia rhodope* and *Bischofia polycarpa* under different climate scenarios

Different Scenarios	Predicted Area of Histia rhodope (10^3 km^2)				Predicted Area of Bischofia polycarpa (10^3 km^2)			
	Lowly suitable habitat	Moderately suitable habitat	Highly suitable habitat	Total Suitability Area	Lowly suitable habitat	Moderately suitable habitat	Highly suitable habitat	Total Suitability Area
Current	763.60	1027.17	373.54	2164.31	546.13	1207.89	400.13	2154.16
2050SSP1-2.6	563.60 (-200)	819.09 (-208)	988.71(615)	2371.40 (207)	468.62 (-77)	753.40 (-454)	1129.10 (729)	2351.11 (197)
2050SSP2-4.5	610.03 (-154)	796.74 (-230)	993.13 (620)	2399.90 (236)	498.30 (-48)	850.14 (-358)	1072.51 (672)	2420.95 (267)
2050SS5-P8.5	642.37 (-121)	816.17 (-211)	1027.83 (654)	2486.37 (322)	518.91 (-27)	801.18 (-407)	1191.76 (792)	2511.86 (358)
2070SSP1-2.6	623.87 (-140)	802.67 (-225)	870.58 (497)	2297.13 (133)	487.23 (-59)	826.16 (-382)	984.14 (584)	2297.52 (143)
2070SSP2-4.5	704.26 (-59)	851.15 (-176)	1034.10 (661)	2589.51 (125)	489.55 (-57)	1115.34 (-93)	1169.88 (770)	2774.77 (621)
2070SSP5-8.5	1049.64 (286)	781.48 (-246)	1343.43 (970)	3174.55 (1010)	597.21 (51)	798.48 (-409)	1222.33 (822)	2618.01 (464)

Note: The numbers in brackets are the difference between the predicted and curren distribition areas.
